# Associations among dietary non-fiber carbohydrate, ruminal microbiota and epithelium G-protein-coupled receptor, and histone deacetylase regulations in goats

**DOI:** 10.1186/s40168-017-0341-z

**Published:** 2017-09-19

**Authors:** Hong Shen, Zhongyan Lu, Zhihui Xu, Zhan Chen, Zanming Shen

**Affiliations:** 1 College of Life Science, Nanjing Agricultural University, Nanjing, Jiangsu China; 2 Bioinformatics Center, Nanjing Agricultural University, Nanjing, Jiangsu China; 3 Key Lab of Animal Physiology and Biochemistry, College of Veterinary Medicine, Nanjing Agricultural University, Nanjing, Jiangsu China

**Keywords:** Rumen microbiota, G-protein-coupled receptors, Histone deacetylases, Dietary modulation, Epithelium physiology, Microbe–host interactions

## Abstract

**Background:**

Diet-derived short-chain fatty acids (SCFAs) in the rumen have broad effects on the health and growth of ruminants. The microbe-G-protein-coupled receptor (GPR) and microbe-histone deacetylase (HDAC) axes might be the major pathway mediating these effects. Here, an integrated approach of transcriptome sequencing and 16S *rRNA* gene sequencing was applied to investigate the synergetic responses of rumen epithelium and rumen microbiota to the increased intake of dietary non-fiber carbohydrate (NFC) from 15 to 30% in the goat model. In addition to the analysis of the microbial composition and identification of the genes and signaling pathways related to the differentially expressed GPRs and HDACs, the combined data including the expression of HDACs and GPRs, the relative abundance of the bacteria, and the molar proportions of the individual SCFAs were used to identify the significant co-variation of the SCFAs, clades, and transcripts.

**Results:**

The major bacterial clades promoted by the 30% NFC diet were related to lactate metabolism and cellulose degradation in the rumen. The predominant functions of the GPR and HDAC regulation network, under the 30% NFC diet, were related to the maintenance of epithelium integrity and the promotion of animal growth. In addition, the molar proportion of butyrate was inversely correlated with the expression of HDAC1, and the relative abundance of the bacteria belonging to Clostridum_IV was positively correlated with the expression of GPR1.

**Conclusions:**

This study revealed that the effects of rumen microbiota-derived SCFA on epithelium growth and metabolism were mediated by the GPR and HDAC regulation network. An understanding of these mechanisms and their relationships to dietary components provides better insights into the modulation of ruminal fermentation and metabolism in the promotion of livestock production.

**Electronic supplementary material:**

The online version of this article (10.1186/s40168-017-0341-z) contains supplementary material, which is available to authorized users.

## Background

Evidence continues to increase implying that the gastrointestinal (GI) microbiota is the critical contributor to host health and GI homeostasis [1]. The dysbiosis of GI microbiota leads to the pathogenesis of a diverse range of diseases affecting host growth and metabolism. So far, the effects of gut microbiota on the host have been suggested to be achieved, at least in part, through the release of short-chain fatty acids (SCFAs), which are the main bacterial metabolites, followed by the fermentation of dietary carbohydrate in the GI tract [2].

In ruminants, SCFAs produced in the rumen meet 70–80% of the energy requirement for the rumen epithelia, and 50–70% of the energy requirement for the animal, thereby playing important roles in maintaining the energy homeostasis of the host [3]. In addition, SCFAs have also been established to modulate a variety of physiological functions of the rumen epithelium. For example, global gene expression profiling, in a study of bovine rumen epithelium cell cultures, has shown that exogenous butyrate promotes the expression of genes associated with cell growth, signal transduction, and immune responses [4]. By dietary intervention and ruminal butyrate infusion, our in vivo investigations have demonstrated that SCFAs promote the expression of genes involved in epithelium growth and SCFA transport [5, 6].

The ability of SCFAs to modulate physiological processes of the epithelium is well known to depend on two major mechanisms in monogastric animals. The first involves epigenetic modulation. SCFAs, especially butyrate, acetate, and propionate, have been established as intrinsic inhibitors of histone deacetylases (HDACs), promoting gene expression via the inhibition of the HDAC-induced deacetylation of lysine residues within histones [7]. The second mechanism regarding SCFA effects is signaling through G-protein-coupled receptors (GPRs). In mice, the upregulation of GPR43/FFAR2 expression in colonic cells has been demonstrated to enhance the circulating levels of the anorectic hormones peptide tyrosine tyrosine (PYY) and glucagon-like peptide-1 (GLP-1), both of which play important roles in the modulation of gut motility and SCFA absorption [8, 9]. Our previous study in goats has revealed that the upregulated expressions of GPR4 and GPR41 are associated with the upregulated expression of urea transporter (UT-B) in the rumen epithelium, indicating the existence of GPR regulation in the rumen [5].

The composition of rumen microbiota has been adequately demonstrated to be correlated with the concentrations of rumen SCFAs in ruminants. For example, the relatively high ratio of Bacteroidetes to Firmicutes is associated with a relatively high SCFA concentration in the rumen [10–12]. Moreover, composition change is an important strategy for rumen microbiota to adapt to external interventions, such as diet, viral infection, and antibiotics. We therefore speculate that the rumen microbiota modulates the physiological processes of the rumen via SCFA-mediated GPR and HDAC mechanisms during dietary modulation. In the present study, we have collected paired epithelial transcriptome and microbial metagenome data from the rumen of goats receiving different concentration diets and have analyzed the correlations among the expression of GPR and HDAC, individual SCFA (acetate, butyrate, propionate, valerate, isobutyrate, and isovalerate) concentrations, and microbiota compositions in the rumen. By the simultaneous measurement of the responses of rumen microbiota and epithelium to dietary modulation, we have gained better insights into the nature of diet–microbiota–host interactions.

## Methods

### Animals

Six male goats (Boer × Yangtze River Delta White, aged 4 months, 17.97 ± 0.84 kg) were randomly allocated into two groups and received either a diet of 65% hay plus 35% concentrate (containing 30% NFC, MC group, *n =* 3) or a diet of 90% hay plus 10% concentrate (containing 15% NFC, LC group, *n =* 3) (Additional file 1: Table S1). All goats were fed with two equal portions of the designed diet at 0800 and 1700 daily for 28 days. Water was freely available to all of the goats during the experimental period. On day 28, the goats were weighted (the final weights of goats in LC group were 18.93 ± 1.25 kg, and the final weights of goats in MC group were 21.4 ± 1.01 kg) and then sacrificed at a local slaughterhouse. This study was approved by the Animal Care and Use Committee of Nanjing Agricultural University, in compliance with the Regulations for the Administration of Affairs Concerning Experimental Animals [13].

### Sample collection and SCFA determination

On day 28, the ruminal fluid was taken at 0, 2, and 5 h after matinal feeding by using the stomach tube and vacuum pump. The first 40 ml of ruminal content was discarded in the procession of collection to avoid the contamination by the saliva. The following 15 ml of ruminal content was strained through a four-layer cheesecloth and immediately subjected to pH measurement. Subsequently, the ruminal fluid was added by 5% HgCl_2_ and stored at − 20 °C for the determination of SCFA concentrations. On day 28, all goats were slaughtered at 8 h after matinal feeding. Immediately after slaughter, the ruminal content was collected and strained through a four-layer cheesecloth. An aliquot (15 ml) of ruminal fluid was collected and stored at − 20 °C for the extraction of microbial DNA. The remaining fluid, added by 5% HgCl_2_, was stored at − 20 °C for the determination of SCFA concentrations. For each goat, around 10 cm^2^ of rumen tissue from the ventral blind sac was quickly excised and washed repeatedly in ice-cold phosphate-buffered saline (PBS; pH 7.4) until the PBS was clear. The muscle layers were removed, and the epithelium was immediately cut into small pieces (around 0.5 cm^2^/piece) and added by TRIzol buffer (Thermo Fisher Scientific, Nanjing, China). These samples were transferred into liquid N within 5 min and stored at − 80 °C until RNA extraction.

The ruminal SCFA concentrations were determined by using a chromatograph (HP6890N, Agilent Technologies, Wilmington, DE, USA) as described in Yang et al. [14]. The SCFA concentrations at 0, 2, 5, and 8 h were triplicate measured, respectively.

### Microbial DNA extraction and sequencing

The metagenomic DNA of the microbiota was extracted from 15 ml ruminal fluid by using a Bacterial DNA Kit (Omega, Shanghai, China). The DNA concentration was determined by means of a Nanodrop 1000 (Thermo Fisher Scientific, Wilmington, DE, USA) and stored at − 20 °C until further processing. The amplicon library preparation was performed by polymerase chain reaction (PCR) amplification of the V3–V4 region of the 16S *rRNA* gene. The universal primers 338F (5′-ACTCCTACGGGAGGCAGCAG-3′) and 806R (5′-GGACTACHVGGGTWTCTAAT-3′) [15], including TruSeq adapter sequences and indices, were used in the PCRs. All libraries were sequenced by using an Illumina MiSeq platform (Illumina, San Diego, CA, USA) at Biomarker Technologies, Beijing, China.

### Ruminal microbiota analysis

Paired reads were filtered for quality (Q30) and joined by FLASH version 1.2.11 [16]. Sequences that contained read lengths shorter than 400 bp were removed. The remaining sequences were then classified into taxa by blasting with the ribosomal database project (RDP) database at a 97% similarity threshold. Operational taxonomic units (OTUs), whose counts were more than 3 in at least one of the samples, were retained in the further analysis. The selected OTUs were normalized to the relative abundance for each sample. The diversity of the microbial communities was estimated by using the R program phyloseq package [17]. For a deeper analysis of the diversity of the major evolutional clades in the ruminal microbiota, OTUs were filtered to acquire a relative abundance of at least 1% in at least one sample. Then, MUSCLE version 3.8.31 [18] was used to align the complete 16S *rRNA* sequences of the corresponding species in the RDP database, and RAxML version 8 [19] and the GTR model were used to construct the maximum likelihood (ML) trees. The R program ape package [20] was used to plot the tree.

### Epithelial RNA extraction and sequencing

Ball mill MM 400 (RETSCH, Germany) was used to homogenize the rumen epithelium before RNA extraction, with the parameters of 25 oscillations/s and 1 min. The homogenized tissue was incubated on ice and immediately used for RNA extraction. Total RNA was extracted by using the RNeasy Mini Kit (Qiagen, Shanghai, China) according to the manufacturer’s instructions. RNA was quantified by using a NanoDrop 1000 spectrophotometer, and its integrity was evaluated by using the RNA 6000 Assay Kit of the Agilent Bioanalyzer 2100 system (Agilent Technologies, CA, USA). High-quality RNA (RNA integrity number > 9.0) was processed by using NEB Next Ultra RNA Library Prep Kit (NEB, USA) following the manufacturer’s instruction. All libraries were sequenced via paired-end chemistry (PE125) on an Illumina HiSeq2500 platform (Illumina, San Diego, CA, USA) at Biomarker Technologies, Beijing, China.

### Transcriptome assembling and differentially expressed gene identification

Low-quality reads were first removed by using an in-house perl script (reads including low-quality bases (Q30) of more than 50% and ambiguous bases (N) of more than 10% were removed). Remaining reads were aligned to the NCBI goat genome annotation release version 101 by using TopHat v2.1.0 [21] with standard parameters. Mapped reads were used to estimate the gene expression level of each gene transcript. Gene expression values were then normalized according to the reads per kilobase million (RPKM). In this study, only genes with at least one RPKM in at least one of the samples were considered as expressed. Each SAM output file from the TopHat alignment was used in the Cuffdiff program of the Cufflinks version 2.2.1 [22] as input files to test for differential gene expression.

### Sequence conservation of differentially expressed HDACs and GPRs

The mRNA sequences of differentially expressed GPRs and HDACs were blasted to the GenBank nucleotide collection, locally. The matched genes, with *e* value less than 1E−5, were pulled out and aligned in MUSCLE version 3.8.31. The maximum likelihood trees of each investigated GPR and HDAC were constructed by using the GTR model in RAxML version 8. Visualization of the phylogenetic trees was performed on the ITOL web server [23].

### HDAC and GPR co-expression network

The co-expressed genes were identified by computing the spearman correlation coefficient (SCC) between pair of genes across six samples. Only expressed genes (RPKM > 1 in at least one sample) were used in the correlation analysis. A threshold for the SCC larger than 0.85 and *p* value less than 0.01 were used to identify significantly co-expressed genes. The expression levels of the first neighbors of investigated HDACs and GPRs on the co-expression network were compared between groups. Only differentially expressed neighbors were further analyzed of Kyoto Encyclopedia of Genes and Genomes (KEGG) pathway on KABAS version 2.0 web server [24]. Finally, cytoscape version 3.4.0 [25] was applied to visualize the gene co-regulation network.

### Dimensionality reduction for microbial features and multidata integration

In order to improve power to associate microbial composition with host gene transcriptional activity, we reduced the dimensionality of microbial features through calculating their SCC with the molar proportions of individual SCFAs, since the molar proportions of SCFAs were stable across the time within the group. The OTUs, whose SCC is larger than 0.6 and *p* value is less than 0.05 with at least one kind of SCFAs, were used in the following analysis. Next, the relationships of the relative abundance of the selected OTUs, the molar proportions of individual SCFAs, and the expressions of the differentially expressed HDACs and GPRs were explored by using the constrained correspondence analysis (CCA) in the R program vegan package [26]. The molar proportions of SCFAs and expression (RPKM) of investigated HDACs and GPRs were used as the environmental factors in the CCA analysis. The R program ggplot2 package [27] was used to generate the visual interpretation of the gene–SCFA–microbiota relationships. The location of each microbial genus stands for the centroid of corresponding OTUs within investigated genus.

## Results

### Microbial metabolites in rumen

**Fig. 1:**
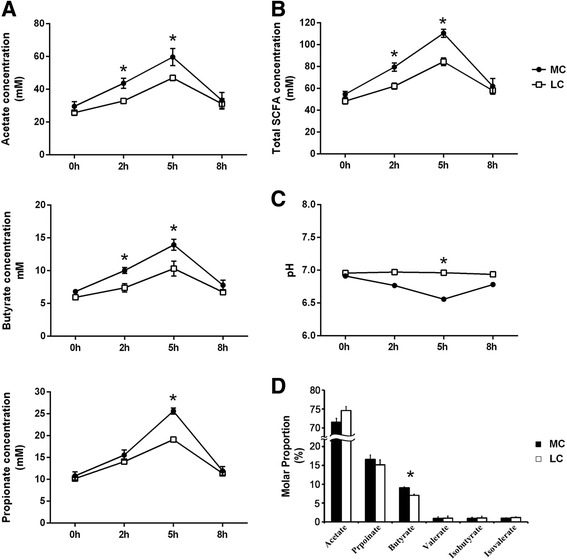
**a** Comparisons of the concentrations of individual SCFAs (acetate, propionate, and butyrate) between the groups. **b** Comparisons of the concentrations of total SCFA between the groups. **c** Comparisons of the concentrations of rumen pH between the groups. **d** Comparisons of the molar proportions of the individual SCFAs between the groups. The molar proportions of the individual SCFAs for ruminal fluid sampled at 8 h after matinal feeding were presented here; 0 h indicates the sampling time just before matinal feeding, and other numbers indicate the sampling time after matinal feeding. Asterisk indicates a *p* value < 0.05 in the *t* test

One-way analysis of variance (ANOVA), included in the SPSS version 13.0.1 (IBM SPSS, Chicago, USA), was performed to compare the concentrations of the individual SCFAs at various time points within the groups. Before matinal feeding (0 h), the concentration of total SCFA and individual SCFAs did not differ between the MC and LC groups (*p* > 0.05) (Fig. 1a, b). Compared with the LC group, at 2 and 5 h after feeding, the concentrations of acetate, butyrate, and total SCFA of the MC group increased significantly (*p* < 0.05) and, thereafter, returned to their pre-feeding levels (basal lines). At 5 h after feeding, the propionate concentration and ruminal pH were significantly higher in the MC group than those in the LC group (*p* < 0.05). But they did not show this significant difference at other time points (*p* > 0.05) (Fig. 1c). At all of the time points, the concentrations of valerate, isobutyrate, and isovalerate were not significantly different between the groups (data not show). Subsequently, the molar proportions of individual SCFAs were compared between and within the groups. Between the groups, the molar proportion of butyrate was significantly higher in the MC group (*p* < 0.05). However, others did not show the significant differences between the groups (*p* > 0.05) (Fig. 1d). The molar proportions of individual SCFAs did not show significantly difference across time (*p* > 0.05, data not show).

### Structure of bacterial communities

**Fig. 2:**
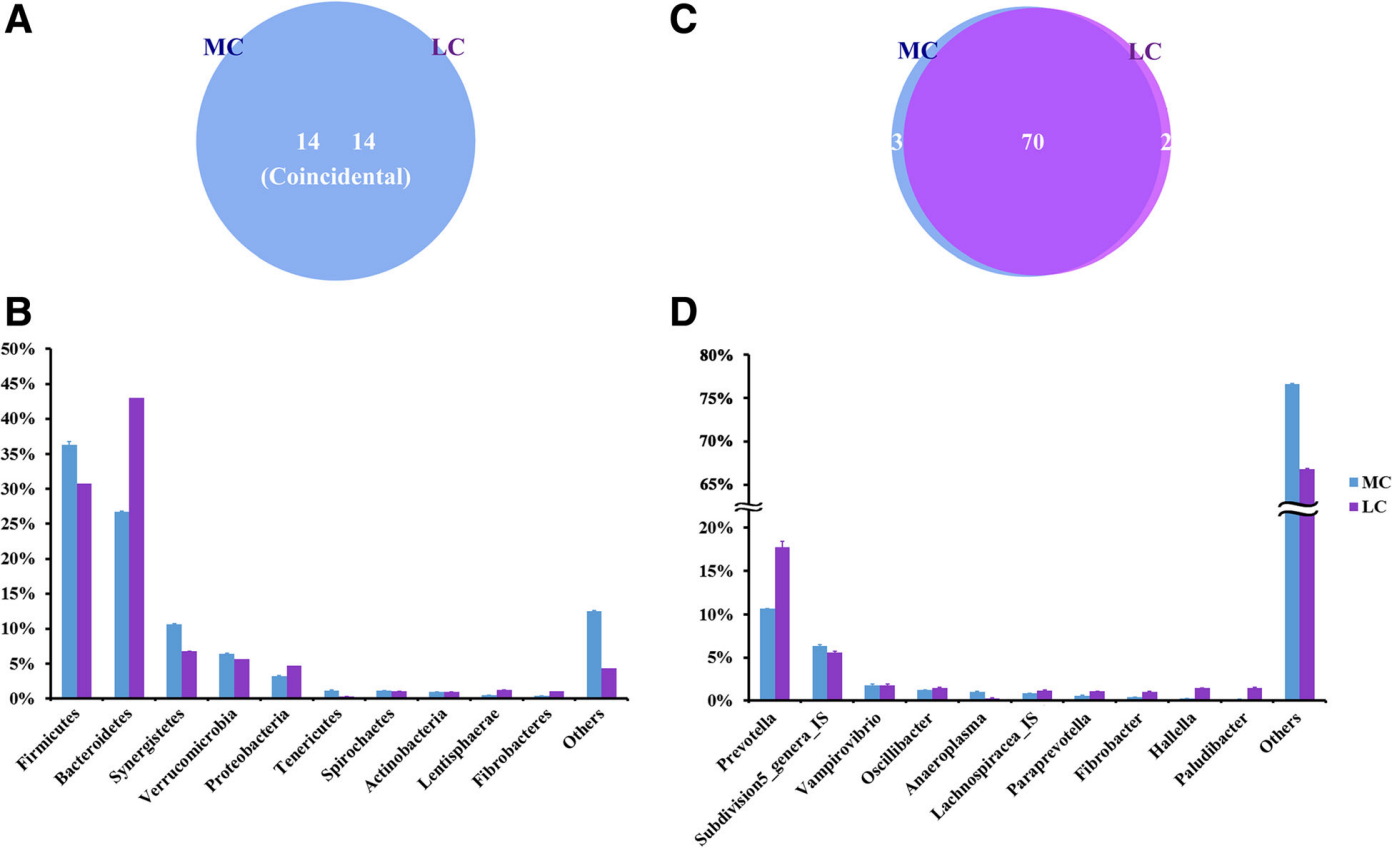
**a** Venn diagram showing the coincidence of phyla between the groups. **b** Phylum-level comparison of bacterial OTUs between the groups. **c** Venn diagram showing the overlap of genera between groups. **d** Genus-level comparison of bacterial OTUs between the groups

At the phylum level, a total of 14 prokaryotic phyla were identified at a 97% similarity, and all of them were common to both groups (Fig. 2a). Firmicutes (35.7–30.1%), Bacteroidetes (26.6–43.6%), and Synergistetes (11.0–7.0%) were the most abundant among all microbial communities. Compared with the LC group, the relative abundance of Synergistetes was increased by 57%, and Tenericutes was increased by 330% in the MC group. They were the two most increased phyla within the microbial communities in MC group. On the contrary, the relative abundance of Lentisphaerae decreased by 63%, and the relative abundance of Fibrobacteres decreased by 65% in the MC group. These were the two most reduced phyla in the microbial communities of the MC group. At the genus level, other than the unclassified OTUs, 75 genera in total were detected in the sequences. Among them, 70 genera were common to both groups (Fig. 2b). The abundances of all genera in both groups are shown in Additional file 2: Table S2. *Prevotella* (10.4–17.9%) was consistently abundant in both groups. Four genera were only detected in the LC group, whereas one genus was only detected in the MC group. Nonmetric multidimensional scaling (NMDS) plot (Additional file 3: Fig. S1) and analysis of similarities (ANOSIM) (*p* < 0.05) revealed the divergence of the community structure in the MC and LC groups.

### Diversity and richness of microbial communities

**Fig. 3:**
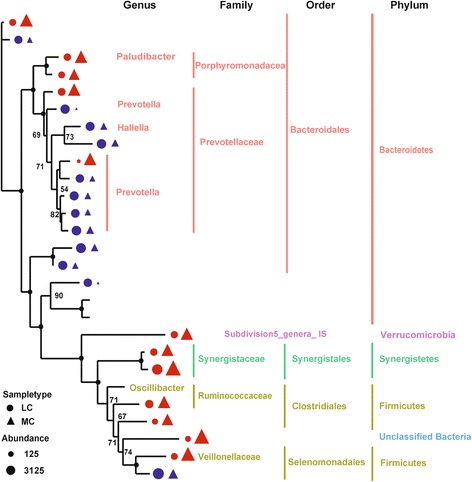
Maximum likelihood tree of 27 detectable OTUs (the relative abundance > 1% in the given sample). The complete 16S *rRNA* gene sequences of the corresponding species in the RDP database were used to construct the tree. Triangle indicates the OTUs in the MC group, and the circle indicates the OTUs in the LC group. Only the OTUs with significant differences (*p* < 0.05) in relative abundance are shown behind the branches. The size of the symbol indicates the relative abundance of OTUs. Red indicates a significant increase (*p* < 0.05) of the relative abundance of the OTU under the 30% NFC diet, and blue indicates a significant reduction (*p* < 0.05) in the relative abundance of the OTU under a 15% NFC diet. Only those bootstrap values greater than 60 are shown on the tree. The solid black circles at the nodes stand for a bootstrap value of 100

No significant difference (*p* > 0.05) was observed on the diversity of the microbial community between the groups, as indicated by the Shannon and Simpson indices. On the phylum level, the diversity of Bacteroidetes and Firmicutes in the MC group was significantly higher than that in the LC group (*p* < 0.05). The diversity of Synergistetes in the MC group was significantly lower than that in the LC group (Additional file 4: Fig. S2). Maximum likelihood (ML) analysis of 27 detectable OTUs (the relative abundance > 1%) showed that the significantly increased OTUs in the MC group belonged to the families Porphyromonadaceae, Ruminococcaceae, Synergistaceae, Veillonellaceae, and Verrucomicrobia subdivision 5 (Fig. 3). On the contrary, 58% (7/12) of the significantly reduced OTUs belonged to the family Prevotellacea.

### Expression profiles of GPRs and HDACs in rumen epithelium

The RNA-Seq method was used to identify the expression profiles of GPRs and HDACs in the rumen of goats. It generated a total of 135 M raw reads (average 22.5 M reads per sample, range 20.9–23.5 M) and 127 M clean reads (average 21.2 M reads per sample, range 19.1–22.5 M). On average, 83% of the clean reads were successfully mapped to the NCBI goat genome annotation release version 101.

The 73 members of the GPR family and the 11 members of the HDAC family were found to be encoded on the goat genome (Additional file 5: Table S3, Additional file 6: Table S4). The transcriptome data showed that 20 members of GPR family and 7 members of HDAC family were expressed in the rumen epithelium (RPKM > 1 in at least one sample). By comparing the gene expressions in the LC group, we found that GPR1, 87, 89A, and 155 were significantly upregulated (*p* < 0.05), and GPR107, free fatty acid receptor 4 (FFAR4, also known as GPR120), and hydroxycarboxylic acid receptor 2 (HCAR2, also known as GPR109A) were significantly downregulated (*p* < 0.05) in the MC group. Moreover, HDAC4, HDAC5, HDAC6, and HDAC10 were significantly upregulated (*p* < 0.05), and HDAC1 was significantly downregulated (*p* < 0.05) in the MC group. By comparing the gene expressions within the group, we found, in both groups, that the expression of GPR87 was the highest among the GPR members, and HDAC1 was the highest among the HDAC members. Further, GPR87 and HDAC1 are the only members that expressed more than 100 counts (RPKM) in at least one group. In addition, six GPRs (GPR1, 89A, 108, 155, 160, and FFAR4) and four HDACs (HDAC2, 3, 5, and 8) expressed more than 10 counts (RPKM) in at least one group. The previously reported GPRs (GPR4, GPR41/FFAR3, GPR43/FFAR2), whose expressions were detected by quantitative PCRs in rumen epithelium [5, 28], were little expressed in the present study.

### Sequence conservation of GPRs and HDACs

To investigate the sequence conservation of differentially expressed GPRs and HDACs during evolution, we constructed ML trees by using all genes found in blast searching, with the threshold of the *e* value less than 1E−5. According to the trees, all the investigated GPRs and HDACs were highly conserved on both of the division and family levels of vertebrates (Additional file 7: Fig. S3, Additional file 8: Fig. S4). To investigate the conservations within the members of GPR family, we pairwise aligned the sequences of encoded GPR members, but no similarity above 30% was received between any pair of GPR members. The same results were received from the comparisons of HDAC members.

### Related KEGG pathways of differentially expressed GPRs and HDACs

**Fig. 4:**
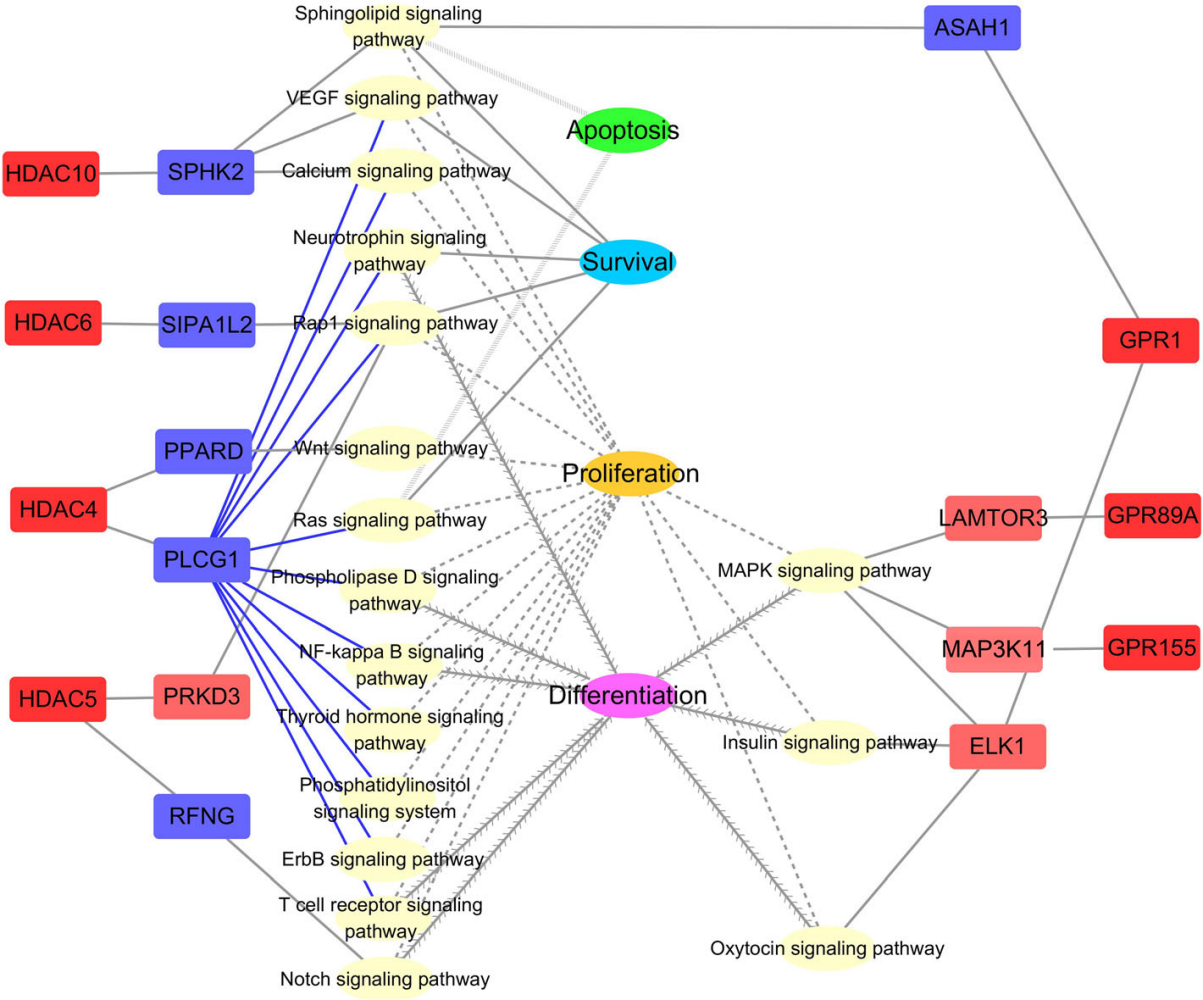
GPR and HDAC co-regulation network related to epithelium growth. The functions of the first neighbors were predicted by the KEGG pathway analysis. The genes colored in red were the genes that were significantly upregulated after 4 weeks of 30% NFC feeding, and the genes colored in blue were the significantly downregulated genes after 4 weeks of 30% NFC feeding. The signaling pathways regulating the same functions are given with the same line shapes in connection with the corresponding functions. Blue lines indicate that the largest number of signaling pathways was regulated by PLCG1

**Fig. 5:**
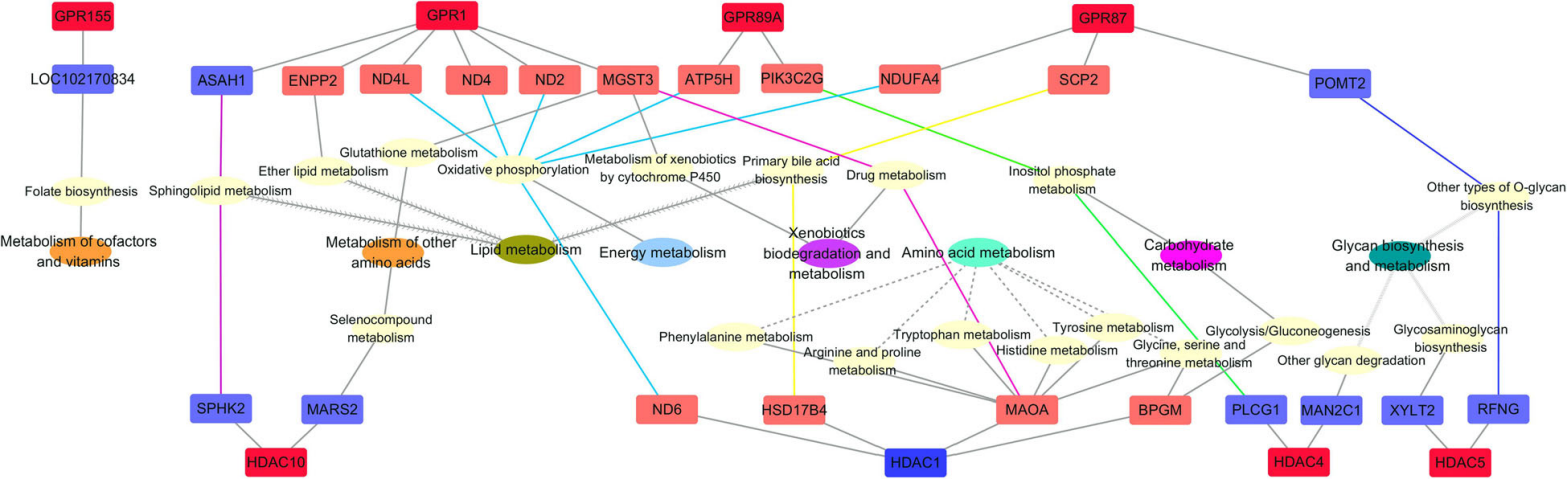
GPR and HDAC co-regulation network related to epithelium metabolism. The functions of the first neighbors were predicted by the KEGG pathway analysis. The genes colored in red were the genes that were significantly upregulated after 4 weeks of 30% NFC feeding, and the genes colored in blue were the significantly downregulated genes after 4 weeks of 30% NFC feeding. The lines in the same color indicate the genes regulating the same metabolic function. The lines in the same shape indicate that the low-level metabolism was grouped in the same high-level metabolism by KEGG annotation

We used SCC to quantify gene–gene co-expression for all of the gene pairs in the transcriptome across the samples. In order to assess the biological significances of the GPR and HDAC co-expression network, we checked the related signaling pathways and possible functions of first neighbors in the KEGG annotation. The received functions of significantly differential neighbors were classified according to the regulated physiological processes of the rumen epithelium. Accordingly, two major kinds of regulation networks were built referred to as the epithelium growth network (including cell apoptosis, survival, proliferation, and differentiation, Fig. 4) and the epithelium metabolism network (including metabolism of cofactors and vitamins, metabolism of other amino acids, lipid metabolism, energy metabolism, xenobiotics biodegradation and metabolism, amino acid metabolism, carbohydrate metabolism, glycan biosynthesis, and metabolism; Fig. 5) in this study. Other possible regulations, involved in the immune responses, transportation, and actin cytoskeleton, are listed in Additional file 9: Table S5.

In the epithelium growth network, the upregulated expressions of GPR1, 89A, and 155 were correlated to the upregulated expressions of regulator complex protein (LAMTOR3), mitogen-activated protein kinase kinase kinase 11 (MAP3K11), and ETS domain-containing protein (ELK1), all of which were located on mitogen-activated protein kinase (MAPK) signaling pathway and related to cell proliferation and differentiation. ELK1 was also on the insulin and oxytocin signaling pathway in the regulation of cell proliferation and differentiation. Moreover, the increased expression of GPR1 was associated with the decreased expression of acid ceramidase (ASAH1), which was on the sphingolipid signaling pathway regulating cell apoptosis, survival, and proliferation. As for HDACs, the upregulation of HDAC 4, 5, 6, and 10 was correlated to the downregulation of five neighbors that regulated the cell apoptosis, survival, proliferation, and differentiation via modulation of 14 signaling pathways.

In the epithelium metabolism network, the upregulation of GPR1, 87, and 89A expression was correlated to the upregulation of nicotinamide adenine dinucleotide dehydrogenase subunits 2, 4, and 4L (ND2, 4, 4L), H^+^ transporting adenosine triphosphate synthase (ATP5H), and nicotinamide adenine dinucleotide dehydrogenase 1 alpha subcomplex 4 (NDUFA4). The downregulation of HDAC1 expression was correlated to the upregulation of ND6 expression. All of these enzymes were related to the oxidative phosphorylation of energy metabolism. The increased expression of GPR1 and decreased expression of HDAC1 were associated with the increased expression of microsomal glutathione S-transferase 3 (MGST3) and monoamine oxidase A (MAOA). These two kinds of enzymes modulated the xenobiotic biodegradation and metabolism of the rumen epithelium. On the contrary, the decreased expressions of protein *O*-mannosyltransferase 2 (POMT2), RFNG *O*-fucosylpeptide 3-beta-*N*-acetylglucosaminyl transferase (RFNG), xylosyltransferase 2 (XYLT2), and mannosidase alpha class 2C member 1 (MAN2C1) were associated with the increased expression of GPR87, HDAC4, and HDAC5. All of these enzymes regulate glycan biosynthesis and metabolism in the rumen epithelium. In addition, the metabolism of other amino acids can be regulated by GPR1-MGST3 and HDAC10-methionyl-tRNA synthetase 2 (MARS2) pathways. Lipid metabolism can be regulated by GPR1-*N*-acylsphingosine amidohydrolase 1 (ASAH1), GPR-ectonucleotide pyrophosphatase 2 (ENPP2), GPR87-propanoyl-CoA *C*-acyltransferase (SCP2), HDAC1-peroxisomal multifunctional enzyme type 2 (HSD17B4), and HDAC10-sphingosine kinase 2 (SPHK2) pathways. Carbohydrate metabolism might be regulated by the GPR89A-phosphatidylinositol-4-phosphate 3-kinase C2 domain-containing gamma polypeptide (PIK3C2G), HDAC4-phospholipase C, gamma 1 (PLCG1), and HDAC1-2,3-bisphosphoglycerate mutase (BPGM) pathways. Amino acid metabolism might be modulated by the HDAC1-MAOA and HDAC1-BPGM pathways. The metabolism of cofactors and vitamins might be modulated by GPR155-molybdenum cofactor biosynthesis protein 1 gene (LOC102170834) pathway.

### Relationships of GPR and HDAC expression, molar proportions of individual SCFAs, and relative abundance of bacterial genera

**Fig. 6:**
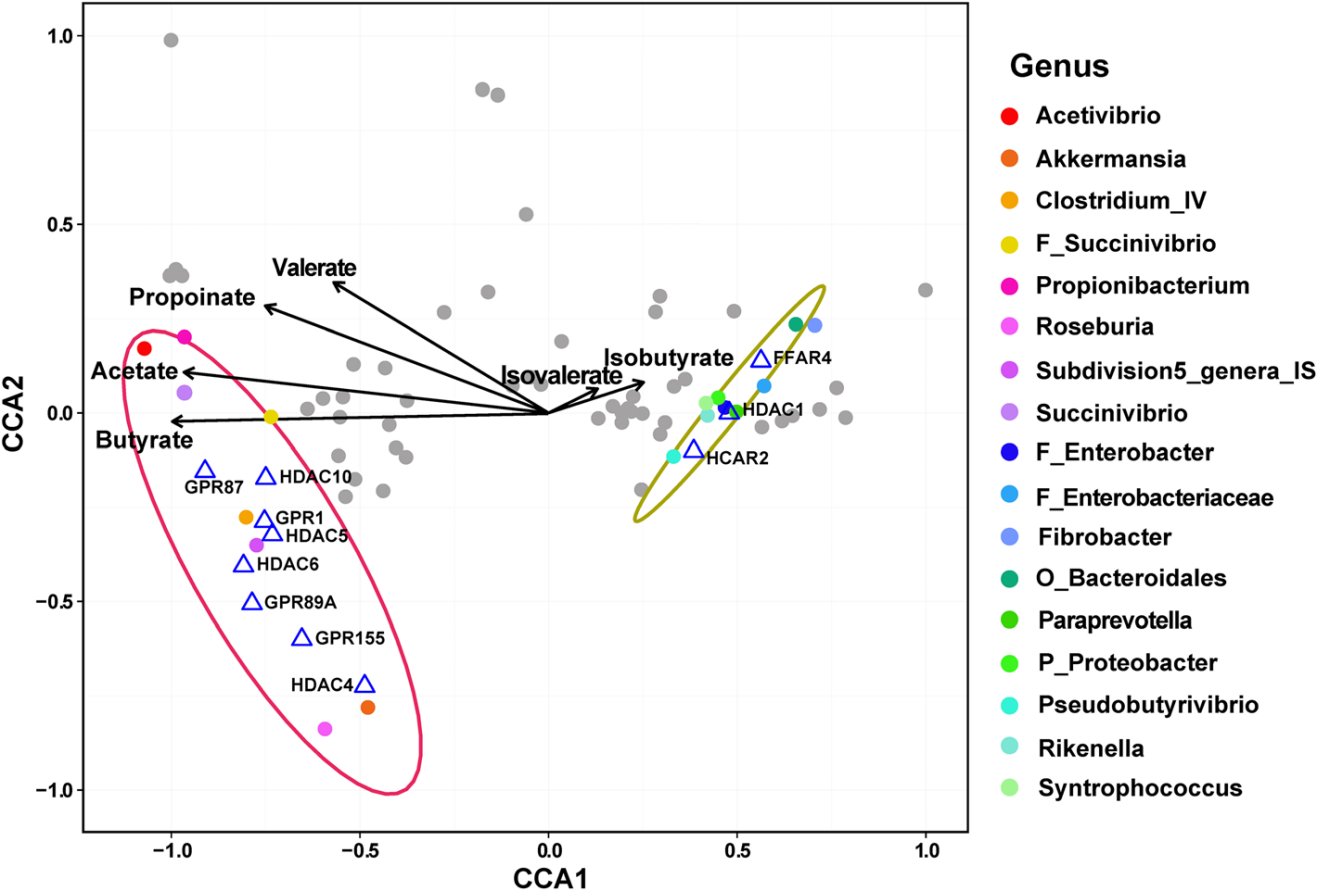
Constrained correspondence analysis revealing the correlations of the relative abundance of the specific microbes (SCC > 0.085 and *p* < 0.01 in a Spearman correlation analysis of the relative abundance of the microbes and the molar proportions of the individual SCFAs), the molar proportions of the individual SCFAs, and the expression of the significantly different HDACs and GPRs

Spearman correlation analysis removed 71% OTUs (611/861), which had SCC less than 0.6 and *p* value greater than 0.05 with any kinds of SCFAs. Subsequently, CCA analysis revealed positive correlations between significantly upregulated GPRs and HDACs, with 8 significantly increased microbial genera (consisting of 56 OTUs), and positive correlations between significantly downregulated GPRs and HDACs, with 9 significantly reduced microbial genera (consisting of 63 OTUs) (Fig. 6). Moreover, the expression of HDAC1 was negatively correlated with the molar proportion of butyrate.

## Discussion

The present study revealed that the MC diet promoted the upregulation of the concentrations of acetate, butyrate, and total SCFA from 2 to 5 h after feeding and the concentration of propionate at 5 h after feeding. Moreover, NMDS and ANOSIM analyses indicated that the changes of ruminal SCFA concentrations were associated with the compositional changes of ruminal microbiota. Phylogenetic analysis of detectable OTUs showed that the relative abundance of five prokaryotic families, namely the Prophyromonadacea, Synergistaceae, Ruminococcaceae, Veillonellaceae, and Verrucomicrobia subdivision 5, were increased significantly in the MC group. Among these taxa promoted by the MC diet, members of the Ruminococcaceae are the important contributors to cellulose degradation [29]. Members of the Veillonellaceae and Prophyromonadacea are capable of utilizing lactate and converting it largely to acetate and propionate [30]. Members of the Synergistaceae are able to ferment amino acids and peptides to produce acetate in animal gut [31]. A recent study of the first cultured representative of Verrucomicrobia subdivision 5 indicated that this strain of the taxon could ferment monosaccharide to produce acetate and lactate [32]. Therefore, we inferred that the bacterial increasing mainly involved the species responsible for decreasing the concentration of lactate in the rumen. Accumulation of lactate in the rumen is a major reason of subacute ruminal acidosis (SARA), characterized by low feed intake and chronic inflammation [10]. Therefore, such reshaping of the microbial community may play important roles in maintaining the stable pH of the rumen during the period of relatively high SCFA concentrations. In addition, the expansion also involved the cellulose-degrading bacteria indicating that the increased dietary NFC in the MC group promoted cellulose degradation by the microbes. This is supported by a previous study in which the positive correlation of the dietary starch and the rate of cellulose degradation were detected [33]. However, the majority of OTUs detected in our investigation have unknown functions, making the interpretation of microbial reshaping associated with SCFA profiles difficult. We can only speculate that the increased butyrate was converted from the acetate by specific microbes via the acetate CoA-transferase pathway, since the expansion of A-producing bacteria and the synergetic upregulation of ruminal butyrate and acetate concentrations were observed.

Broad effects of SCFAs on the physiological processes of ruminal epithelium have been reported. However, little is known concerning the mechanisms related to this SCFA-mediated regulation in the rumen. In present study, we observed the specific expression of the members of the GPR and HDAC families in the rumen epithelium. However, the previously reported GPRs (GPR4, GPR41/FFAR3, GPR43/FFAR2), whose expressions were detected by quantitative PCRs in rumen epithelium [5, 28], were little expressed in the present study. The disturbances of unmapped reads in transcriptome assembling and an overestimation of isoform expressions in quantitative PCRs might be the reasons for the inconsistent results. Since the highly conserved sequences of these genes within vertebrates, our results indicate the possibility that commensal bacteria regulate the epithelium physiology via metabolite-mediated GPR and HDAC pathways in the rumen. Furthermore, our data suggest the GPR87 and HDAC1 play more important roles in the ruminal epithelium than other members of the respective families.

According to our epithelium growth network, in the MC group, GPR1, 89A, and 155 might promote cell proliferation and differentiation via the MAPK, sphingolipid, insulin, and oxytocin signaling pathways. Also in MC group, HDAC 4, 5, 6, and 10 may suppress cell apoptosis, survival, proliferation, and differentiation by suppressing the expression of genes located on 14 signaling pathways. Among them, nuclear factor kappa B signaling has been established to control the expression of cyclin D1 via the modulation of the production of pro-inflammatory cytokines in mice [34, 35]. WNT signaling has been shown to regulate cell differentiation and apoptosis in a dose-dependent manner in human, i.e., low-intensity signaling leads to controlled self-renewal, moderate-intensity signaling promotes uncontrolled cell proliferation, and high-intensity signaling leads to apoptosis [36, 37]. Calcium signaling has also been demonstrated to regulate the cell cycle by decreasing intracellular cyclic adenosine monophosphate (cAMP) in human [38]. Taken together, these data suggest that, in an environment with relatively high SCFA concentrations, GPRs promote cell proliferation and differentiation via the promotion of the expressions of related genes. However, at the same time, HDACs might suppress cell proliferation, differentiation, and survival by suppressing the expression of another set of related genes. Interestingly, the sphingolipid signaling pathway, which is related to cell apoptosis, might be suppressed by both HDAC10 and GPR1 in the MC group. These results suggest that epithelium homeostasis is maintained by GPR and HDAC co-regulation mechanisms.

Our analysis of the epithelium metabolism network has revealed that, in the MC group, the changed expressions of GPR1, 87, 89A, and HDAC1 possibly enhance energy metabolism by promoting the expressions of seven genes related to mitochondrial respiratory chain function. The decreased expression of HDAC1 might enhance glycolysis by promoting the expression of BPGM, a crucial enzyme in the regulation of hemoglobin-associated oxygen. Moreover, the decreased expression of HDAC1 might enhance amino acid metabolism by promoting the expression of MAOA, the enzyme catalyzing the oxidative deamination of monoamines. The enhanced capability of amino acid metabolism by the epithelium might increase ketone synthesis, providing more energy for the animal. In addition, the suppression of glycosaminoglycan biosynthesis and glycan degradation by HDAC5 possibly increases the amount of glycan entering the blood. Promotion of primary bile acid biosynthesis by GPR87 and HDAC1 probably enhances glucose metabolism and energy expenditure via the bile acid signaling pathway [39]. All the abovementioned changes in the epithelium metabolism of the MC group will improve energy supplements for the animal. Additionally, in the MC group, the enhancement of exogenous toxin degradation by GPR1 and HDAC1 benefits the animal health. Suppression of *O*-glycan biosynthesis by GPR87 and HDAC5 might inhibit the activation of T lymphocytes [40]. Suppression of sphingolipid metabolism by GPR1 and HDAC10 possibly promotes the stability of membrane structure and cell-to-cell recognition [41, 42]. Enhancing the ether lipid metabolism by GPR1 can increase the activity of the potassium channel [43], maintaining the stability of the rumen ecosystem. So far, GPRs have been reported to regulate host metabolism via the modulation of hormone secretion, sympathetic activity, and immunity responses [9, 44, 45]. Our results suggest that the regulation of GPRs and HDACs in epithelium metabolism have major effects via the modulation of the expression of related enzymes. Furthermore, we have found that the regulation of GPRs and HDACs, induced by relatively high SCFA concentrations, will benefit animal growth and health.

Studies in humans have shown that gut microbe-derived butyrate was the most potent inhibitor of HDACs, with a maximum efficiency of approximately 80% inhibition of HDAC1/2 [46]. In present study, the highly negative correlation between the molar proportion of butyrate and the expression of HDAC1, revealed by CCA analysis, suggested that, in the goat rumen, microbiota-derived butyrate was also the most powerful inhibitor of HDAC1 in epithelium. In addition, the study of Kimura et al. [45] revealed that ffar2-deficient and ffar2-overexpressing mice exhibited different phenotypes (obesity or leanness, respectively) in comparison with wild-type mice. However, these mice lost the corresponding phenotypes when grown under germ-free conditions or when treated with antibiotics suggesting that the activation of GPRs might be dependent on the existence of gut microbes. In our CCA analysis, a high correlation existed between the relative abundance of Clostridium_IV and the expressions of GPR1. Members of Clostridium_IV are well known to be the major butyrate-producing bacteria in the animal gut [47]. A previous study has shown that the depletion of butyrate-producing bacteria is a common feature in patients suffering from inflammatory bowel diseases (IBD) and colorectal cancer (CRC) [48]. Despite limitations, we speculate that butyrate-producing bacteria play an important role in the promotion of epithelium growth and metabolism via the modulation of the expression of GPR1. Unfortunately, most of the significantly correlated species cannot as yet be cultured, and we cannot infer their roles in these regulation networks. Further studies are required for the identification of their functions and significance in the animal gut.

## Conclusion

Our results indicate that SCFA-mediated GPR and HDAC co-regulation networks exist in the rumen epithelium, allowing the animals to receive signaling from their resident microbiota, sensitively and accurately. These networks regulate a variety of physiological processes in the epithelium, especially growth and metabolism, and play important roles in the maintenance of epithelial integrity and the promotion of animal growth. Moreover, these networks are important mechanisms for commensal bacteria to improve their co-habiting conditions by the modulation of epithelium physiology. By understanding these reciprocal mechanisms between the host and microbiota, coupled with the corresponding modulation factors in the diet, we will be able to improve animal production in healthy and sustainable ways.

## Additional files

Additional file 1: Table S1. Dietary compositions used in this study. (PDF 33 kb)

Additional file 2: Table S2. Rumen microbial compositions of the MC and LC groups. (PDF 43 kb)

Additional file 3: Figure. S1. Nonmetric multidimensional scaling (NMDS) analysis of Bray–Curtis similarity coefficients based on the relative abundance of OTUs in the given sample. (PDF 52 kb)

Additional file 4: Figure. S2. Diversity of the prokaryotic taxa estimated by using the Shannon index. (PDF 128 kb)

Additional file 5: Table S3. Expression profile of the members of the GPR family observed in present study. (PDF 14 kb)

Additional file 6: Table S4. Expression profile of the members of the HDAC family observed in present study. (PDF 10 kb)

Additional file 7: Figure S3. Maximum likelihood trees constructed by using the GPR sequences resulting from blast searching in the NCBI nucleotide collection with the threshold of the *e* value less than 1E−5. (PDF 770 kb)

Additional file 8: Figure S4. Maximum likelihood trees constructed by using the HDAC sequences resulting from blast searching in the NCBI nucleotide collection with the threshold of the *e* value less than 1E−5. (PDF 542 kb)

Additional file 9: Table S5. Other possible functions of the first neighbors connected to the differentially expressed GPRs and HDACs in the network. (PDF 63 kb)

## References

[1] Ohland CL, Jobin C (2015). Microbial activities and intestinal homeostasis: a delicate balance between health and disease. Cell Mol Gastroenterol Hepatol.

[2] Tan J, McKenzie C, Potamitis M, Thorburn AN, Mackay CR, Macia L (2014). The role of short-chain fatty acids in health and disease. Adv Immunol.

[3] Bergman EN (1990). Energy contributions of volatile fatty acids from the gastrointestinal tract in various species. Physiol Rev.

[4] Li CJ, Li RW, Baldwin RL, Blomberg le A, Wu S, Li W (2016). Transcriptomic sequencing reveals a set of unique genes activated by butyrate-induced histone modification. Gene Regul Syst Bio.

[5] Lu Z, Gui H, Yao L, Yan L, Martens H, Aschenbach JR, Shen Z (2015). Short-chain fatty acids and acidic pH upregulate UT-B, GPR41, and GPR4 in rumen epithelial cells of goats. Am J Phys Regul Integr Comp Phys.

[6] Malhi M, Gui H, Yao L, Aschenbach JR, Gabel G, Shen Z (2013). Increased papillae growth and enhanced short-chain fatty acid absorption in the rumen of goats are associated with transient increases in cyclin D1 expression after ruminal butyrate infusion. J Dairy Sci.

[7] MacDonald VE, Howe LJ (2009). Histone acetylation: where to go and how to get there. Epigenetics.

[8] Samuel BS, Shaito A, Motoike T, Rey FE, Backhed F, Manchester JK, Hammer RE, Williams SC, Crowley J, Yanagisawa M, Gordon JI (2008). Effects of the gut microbiota on host adiposity are modulated by the short-chain fatty-acid binding G protein-coupled receptor, Gpr41. Proc Natl Acad Sci U S A.

[9] Tolhurst G, Heffron H, Lam YS, Parker HE, Habib AM, Diakogiannaki E, Cameron J, Grosse J, Reimann F, Gribble FM (2012). Short-chain fatty acids stimulate glucagon-like peptide-1 secretion via the G-protein-coupled receptor FFAR2. Diabetes.

[10] Mao SY, Huo WJ, Zhu WY (2016). Microbiome-metabolome analysis reveals unhealthy alterations in the composition and metabolism of ruminal microbiota with increasing dietary grain in a goat model. Environ Microbiol.

[11] Mohammadzadeh H, Yanez-Ruiz DR, Martinez-Fernandez G, Abecia L (2014). Molecular comparative assessment of the microbial ecosystem in rumen and faeces of goats fed alfalfa hay alone or combined with oats. Anaerobe.

[12] Zhang R, Zhu W, Zhu W, Liu J, Mao S (2014). Effect of dietary forage sources on rumen microbiota, rumen fermentation and biogenic amines in dairy cows. J Sci Food Agric.

[13] China SCoPR (1988). Administration of affairs concerning experimental animals.

[14] Yang W, Shen Z, Martens H (2012). An energy-rich diet enhances expression of Na(+)/H(+) exchanger isoform 1 and 3 messenger RNA in rumen epithelium of goat. J Anim Sci.

[15] Mori H, Maruyama F, Kato H, Toyoda A, Dozono A, Ohtsubo Y, Nagata Y, Fujiyama A, Tsuda M, Kurokawa K (2014). Design and experimental application of a novel non-degenerate universal primer set that amplifies prokaryotic 16S rRNA genes with a low possibility to amplify eukaryotic rRNA genes. DNA Res.

[16] Magoc T, Salzberg SL (2011). FLASH: fast length adjustment of short reads to improve genome assemblies. Bioinformatics.

[17] McMurdie PJ, Holmes S (2013). Phyloseq: an R package for reproducible interactive analysis and graphics of microbiome census data. PLoS One.

[18] Edgar RC (2004). MUSCLE: a multiple sequence alignment method with reduced time and space complexity. BMC Bioinformatics.

[19] Stamatakis A (2014). RAxML version 8: a tool for phylogenetic analysis and post-analysis of large phylogenies. Bioinformatics.

[20] Paradis E, Claude J, Strimmer K (2004). APE: analyses of phylogenetics and evolution in R language. Bioinformatics.

[21] Kim D, Pertea G, Trapnell C, Pimentel H, Kelley R, Salzberg SL (2013). TopHat2: accurate alignment of transcriptomes in the presence of insertions, deletions and gene fusions. Genome Biol.

[22] Trapnell C, Williams BA, Pertea G, Mortazavi A, Kwan G, van Baren MJ, Salzberg SL, Wold BJ, Pachter L (2010). Transcript assembly and quantification by RNA-Seq reveals unannotated transcripts and isoform switching during cell differentiation. Nat Biotechnol.

[23] Letunic I, Bork P (2016). Interactive tree of life (iTOL) v3: an online tool for the display and annotation of phylogenetic and other trees. Nucleic Acids Res.

[24] Xie C, Mao X, Huang J, Ding Y, Wu J, Dong S, Kong L, Gao G, Li CY, Wei L (2011). KOBAS 2.0: a web server for annotation and identification of enriched pathways and diseases. Nucleic Acids Res.

[25] Shannon P, Markiel A, Ozier O, Baliga NS, Wang JT, Ramage D, Amin N, Schwikowski B, Ideker T (2003). Cytoscape: a software environment for integrated models of biomolecular interaction networks. Genome Res.

[26] Oksanen J, Blanchet GF, Friendly M, Kindt R, Legendre P, McGlinn D, Minchin PR, O'Hara RB, Simpson GL, Solymos P (2016). Vegan: community ecology package.

[27] Wickham H (2009). ggplot2: elegant graphics for data analysis.

[28] Wang A, Gu Z, Heid B, Akers RM, Jiang H (2009). Identification and characterization of the bovine G protein-coupled receptor GPR41 and GPR43 genes. J Dairy Sci.

[29] Weimer PJ (1996). Why don’t ruminal bacteria digest cellulose faster?. J Dairy Sci.

[30] Duncan SH, Barcenilla A, Stewart CS, Pryde SE, Flint HJ (2002). Acetate utilization and butyryl coenzyme a (CoA):acetate-CoA transferase in butyrate-producing bacteria from the human large intestine. Appl Environ Microbiol.

[31] Leong LE, Denman SE, Hugenholtz P, McSweeney CS (2016). Amino acid and peptide utilization profiles of the fluoroacetate-degrading bacterium Synergistetes strain MFA1 under varying conditions. Microb Ecol.

[32] Spring S, Bunk B, Sproer C, Schumann P, Rohde M, Tindall BJ, Klenk HP (2016). Characterization of the first cultured representative of Verrucomicrobia subdivision 5 indicates the proposal of a novel phylum. ISME J.

[33] Han Z, Chen J (1988). Rumen digestion and metabolism.

[34] Inan MS, Rasoulpour RJ, Yin L, Hubbard AK, Rosenberg DW, Giardina C (2000). The luminal short-chain fatty acid butyrate modulates NF-kappaB activity in a human colonic epithelial cell line. Gastroenterology.

[35] Segain JP, Raingeard de la Bletiere D, Bourreille A, Leray V, Gervois N, Rosales C, Ferrier L, Bonnet C, Blottiere HM, Galmiche JP (2000). Butyrate inhibits inflammatory responses through NFkappaB inhibition: implications for Crohn’s disease. Gut.

[36] Bordonaro M, Lazarova DL, Sartorelli AC (2008). Hyperinduction of Wnt activity: a new paradigm for the treatment of colorectal cancer?. Oncol Res.

[37] Lazarova DL, Bordonaro M, Carbone R, Sartorelli AC (2004). Linear relationship between Wnt activity levels and apoptosis in colorectal carcinoma cells exposed to butyrate. Int J Cancer.

[38] Yonezawa T, Haga S, Kobayashi Y, Katoh K, Obara Y (2008). Unsaturated fatty acids promote proliferation via ERK1/2 and Akt pathway in bovine mammary epithelial cells. Biochem Biophys Res Commun.

[39] Taoka H, Yokoyama Y, Morimoto K, Kitamura N, Tanigaki T, Takashina Y, Tsubota K, Watanabe M (2016). Role of bile acids in the regulation of the metabolic pathways. World J Diabetes.

[40] Priatel JJ, Chui D, Hiraoka N, Simmons CJ, Richardson KB, Page DM, Fukuda M, Varki NM, Marth JD (2000). The ST3Gal-I sialyltransferase controls CD8+ T lymphocyte homeostasis by modulating O-glycan biosynthesis. Immunity.

[41] Merrill AH (2002). De novo sphingolipid biosynthesis: a necessary, but dangerous, pathway. J Biol Chem.

[42] Zheng W, Kollmeyer J, Symolon H, Momin A, Munter E, Wang E, Kelly S, Allegood JC, Liu Y, Peng Q (2006). Ceramides and other bioactive sphingolipid backbones in health and disease: lipidomic analysis, metabolism and roles in membrane structure, dynamics, signaling and autophagy. Biochim Biophys Acta.

[43] Berthe W, Sevrain CM, Chantome A, Bouchet AM, Gueguinou M, Fourbon Y, Potier-Cartereau M, Haelters JP, Couthon-Gourves H, Vandier C, Jaffres PA (2016). New disaccharide-based ether lipids as SK3 ion channel inhibitors. ChemMedChem.

[44] De Vadder F, Kovatcheva-Datchary P, Goncalves D, Vinera J, Zitoun C, Duchampt A, Backhed F, Mithieux G (2014). Microbiota-generated metabolites promote metabolic benefits via gut-brain neural circuits. Cell.

[45] Kimura I, Ozawa K, Inoue D, Imamura T, Kimura K, Maeda T, Terasawa K, Kashihara D, Hirano K, Tani T (2013). The gut microbiota suppresses insulin-mediated fat accumulation via the short-chain fatty acid receptor GPR43. Nat Commun.

[46] Kasubuchi M, Hasegawa S, Hiramatsu T, Ichimura A, Kimura I (2015). Dietary gut microbial metabolites, short-chain fatty acids, and host metabolic regulation. Nutrients.

[47] Barcenilla A, Pryde SE, Martin JC, Duncan SH, Stewart CS, Henderson C, Flint HJ (2000). Phylogenetic relationships of butyrate-producing bacteria from the human gut. Appl Environ Microbiol.

[48] Guzman JR, Conlin VS, Jobin C (2013). Diet, microbiome, and the intestinal epithelium: an essential triumvirate?. Biomed Res Int.

