# Supplementary material for: Associations among dietary non-fiber carbohydrate, ruminal microbiota and epithelium G-protein-coupled receptor, and histone deacetylase regulations in goats
Source: Microbiome. 2017 Sep 19;5:123. doi: 10.1186/s40168-017-0341-z (PMC5606034; doi:10.1186/s40168-017-0341-z)
Supplement: Supplementary file 1 — Dietary compositions used in this study. (PDF 33 kb) [file 40168_2017_341_MOESM1_ESM.pdf]

Table S1 Dietary compositions used in this study[1]

Item	MC[2]	LC[2]
**Dietary intake**	guinea grass + corn	guinea grass
Total DMI[3], g/d	773.61	783.3
NDF[4], g/d	414.66	527.07
NFC[5, 6], g/d	218.71	110.31
ME, kJ/(kg$^{0.75}$·d)	738.05	710.53
**Ingredient, % of DM**		
Guinea Grass	65	90
Corn	25	0
Soya bean meal	8	8
Additive[7]	2	2
**Chemical composition**		
DM,%	89.46	90.63
Crude protein, %DM	9.98	9.61
Crude fat, %DM	2.94	3
Crude fibre, %DM	22.84	29.51
Crude ash, %DM	5.2	6.02
NDF, %DM	28.27	14.08
NFC, %DM	53.6	67.29
ME, mJ/kg DM	5.99	5.63

[1]The values are means ± SEM.

[2] MC (*n* = 3), LC (*n* = 3)

[3] DMI: dry matter intake; DM: dry matter

[4] NDF: neutral detergent fibre

[5] NFC: non fibrous carbohydrate

[6] NFC = 100 – (NDF + CP + crude fat + ash)

[7] The additive was composed of calcium phosphate, limestone, trace mineral salt, and vitamin premix (vitamins A, D, and E).
