# Supplementary material for: Associations among dietary non-fiber carbohydrate, ruminal microbiota and epithelium G-protein-coupled receptor, and histone deacetylase regulations in goats
Source: Microbiome. 2017 Sep 19;5:123. doi: 10.1186/s40168-017-0341-z (PMC5606034; doi:10.1186/s40168-017-0341-z)
Supplement: Supplementary file 2 — Rumen microbial compositions of the MC and LC groups. (PDF 43 kb) [file 40168_2017_341_MOESM2_ESM.pdf]

Table S2. Rumen microbial compositions of the MC and LC groups

Item	Genus	MC	LC
1	F_Synergistaceae	11029±136	6982±44
2	Prevotella	10409±51	17949±687
3	F_Veillonellaceae	6629±177	2984±93
4	Subdivision5_genera_IS	6513±105	5711±142
5	F_Ruminococcaceae	6295±141	5301±116
6	F_Lachnospiraceae	5827±48	3441±52
7	F_Porphyromonadaceae	4867±171	1418±28
8	O_Clostridiales	4635±84	2479±41
9	O_Bacteroidales	4159±61	10386±164
10	P_Firmicutes	3479±57	2225±124
11	P_Bacteroidetes	3013±42	4709±112
12	F_Prevotellaceae	2811±87	4163±79
13	Vampirovibrio	1822±78	1811±89
14	O_Selenomonadales	1494±40	6920±61
15	Oscillibacter	1309±8	1504±51
16	F_Erysipelotrichaceae	1051±48	150±11
17	Anaeroplasma	1048±76	286±17
18	Clostridium_IV	823±58	168±17
19	Lachnospiracea_IS	814±20	1187±49
20	Butyrivibrio	795±28	693±33
21	Saccharibacteria_genera_IS	781±53	834±33
22	Anaerovorax	712±45	888±43
23	Treponema	582±19	871±36
24	Paraprevotella	563±42	1114±23
25	F_Coriobacteriaceae	549±32	486±13
26	Ruminococcus	512±47	485±41
27	F_Desulfovibrionaceae	438±13	176±29
28	Barnesiella	378±26	458±38
29	Fibrobacter	364±35	1038±64
30	C_Alphaproteobacteria	363±21	1541±77
31	Victivallis	310±12	945±20
32	Schwartzia	304±35	322±15
33	Sphaerochaeta	302±14	38±1
34	Acetivibrio	257±17	302±19
35	F_Spirochaetaceae	254±21	191±28
36	Hallella	247±16	1504±18
37	F_Anaerolineaceae	211±25	48±15
38	Slackia	208±21	380±16
39	Paludibacter	183±45	1534±58
40	F_Mycoplasmataceae	182±21	0
41	Oligosphaera	169±21	321±25
42	F_Enterobacteriaceae	157±28	310±26

43	C_Clostridia	138±13	57±8
44	Syntrophococcus	126±13	215±17
45	C_Deltaproteobacteria	121±15	439±25
46	O_Campylobacterales	118±11	99±12
47	Saccharofermentans	106±19	246±32
48	Clostridium_XlVa	104±24	70±6
49	Desulfovibrio	80±4	93±16
50	Roseburia	70±8	105±13
51	Moryella	64±10	28±2
52	Olsenella	51±3	17±6
53	Anaerovibrio	50±4	31±8
54	Succiniclasticum	47±7	38±17
55	Atopobium	45±10	58±0
56	Sporobacter	40±7	149±2
57	Coprococcus	39±5	44±8
58	O_Desulfuromonadales	38±7	33±11
59	Howardella	37±6	28±3
60	Candidatus_Endomicrobium	37±4	26±7
61	Pseudobutyrivibrio	19±7	52±10
62	Enterorhabdus	18±6	13±4
63	P_Proteobacteria	15±7	32±9
64	Armatimonadetes_gp2	13±9	94±11
65	Blautia	12±3	0
66	Mogibacterium	9±6	18±7
67	Suttonella	7±4	21±12
68	F_Puniceicoccaceae	4±4	15±7
69	F_Sphingobacteriaceae	4±4	252±15
70	F_Sutterellaceae	3±3	41±6
71	Bilophila	3±3	23±5
72	F_Peptostreptococcaceae	0	15±5
73	Akkermansia	0	17±4
74	Alloprevotella	0	64±3
75	Unclassfied_Bacetria	11741±154	3302±93

Values are mean ±standard error of mean (SEM).

OTUs less than 3 counts in all samples were removed, and then normalized to the relative abundance for each sample (showed here in a scale of 1/100,000).

Genera were sorted based on their relative abundance in a descending order.

F = Family; O = Order; C = Class; P = Phylum.
