# Supplementary material for: Associations among dietary non-fiber carbohydrate, ruminal microbiota and epithelium G-protein-coupled receptor, and histone deacetylase regulations in goats
Source: Microbiome. 2017 Sep 19;5:123. doi: 10.1186/s40168-017-0341-z (PMC5606034; doi:10.1186/s40168-017-0341-z)

Fig. S1. Nonmetric multidimensional scaling (NMDS) analysis of Bray-Curtis similarity coefficients based on the relative abundance of OTUs in the given sample.

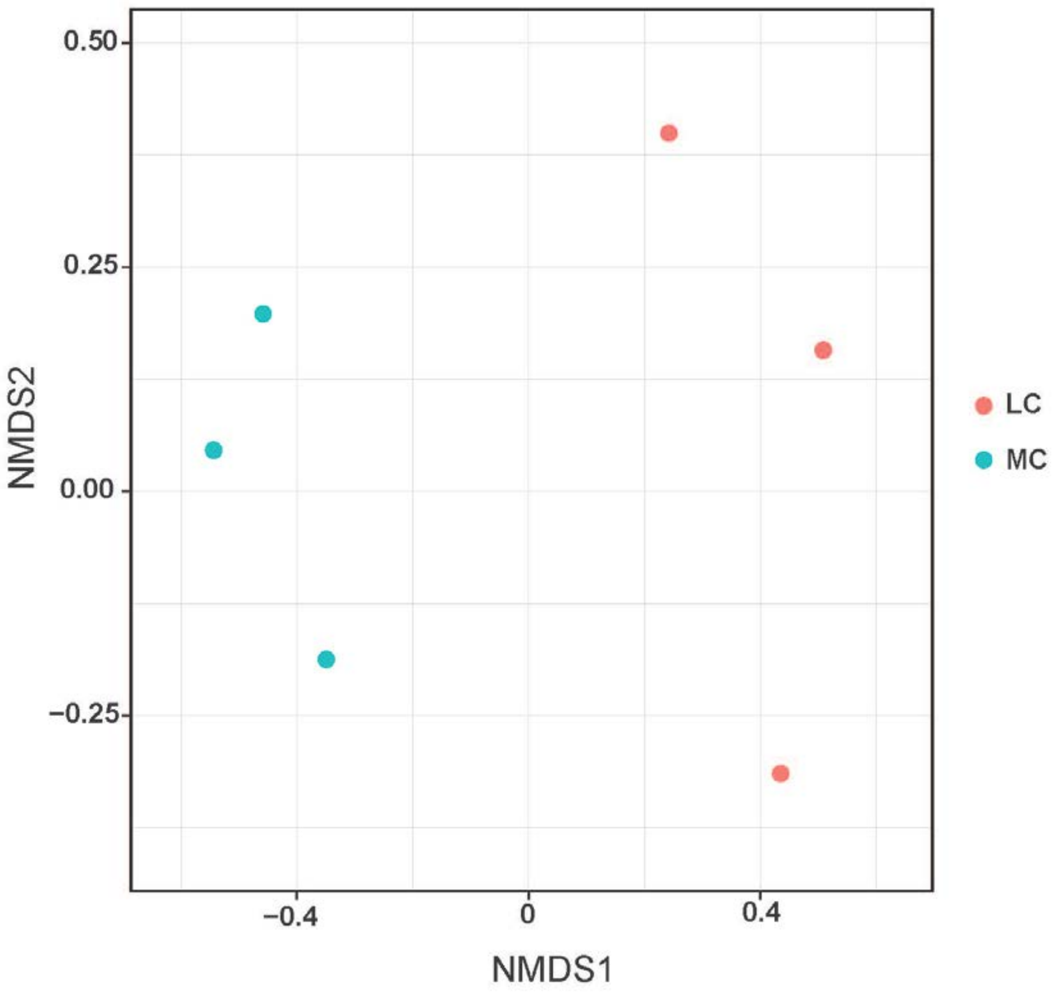

Supplement: Supplementary file 3 — Nonmetric multidimensional scaling (NMDS) analysis of Bray–Curtis similarity coefficients based on the relative abundance of OTUs in the given sample. (PDF 52 kb) [file 40168_2017_341_MOESM3_ESM.pdf]
