# Supplementary figures and images for: Associations among dietary non-fiber carbohydrate, ruminal microbiota and epithelium G-protein-coupled receptor, and histone deacetylase regulations in goats
Source: Microbiome. 2017 Sep 19;5:123. doi: 10.1186/s40168-017-0341-z (PMC5606034; doi:10.1186/s40168-017-0341-z)

Fig. S2. Diversity of the prokaryotic taxa estimated by using the Shannon index.

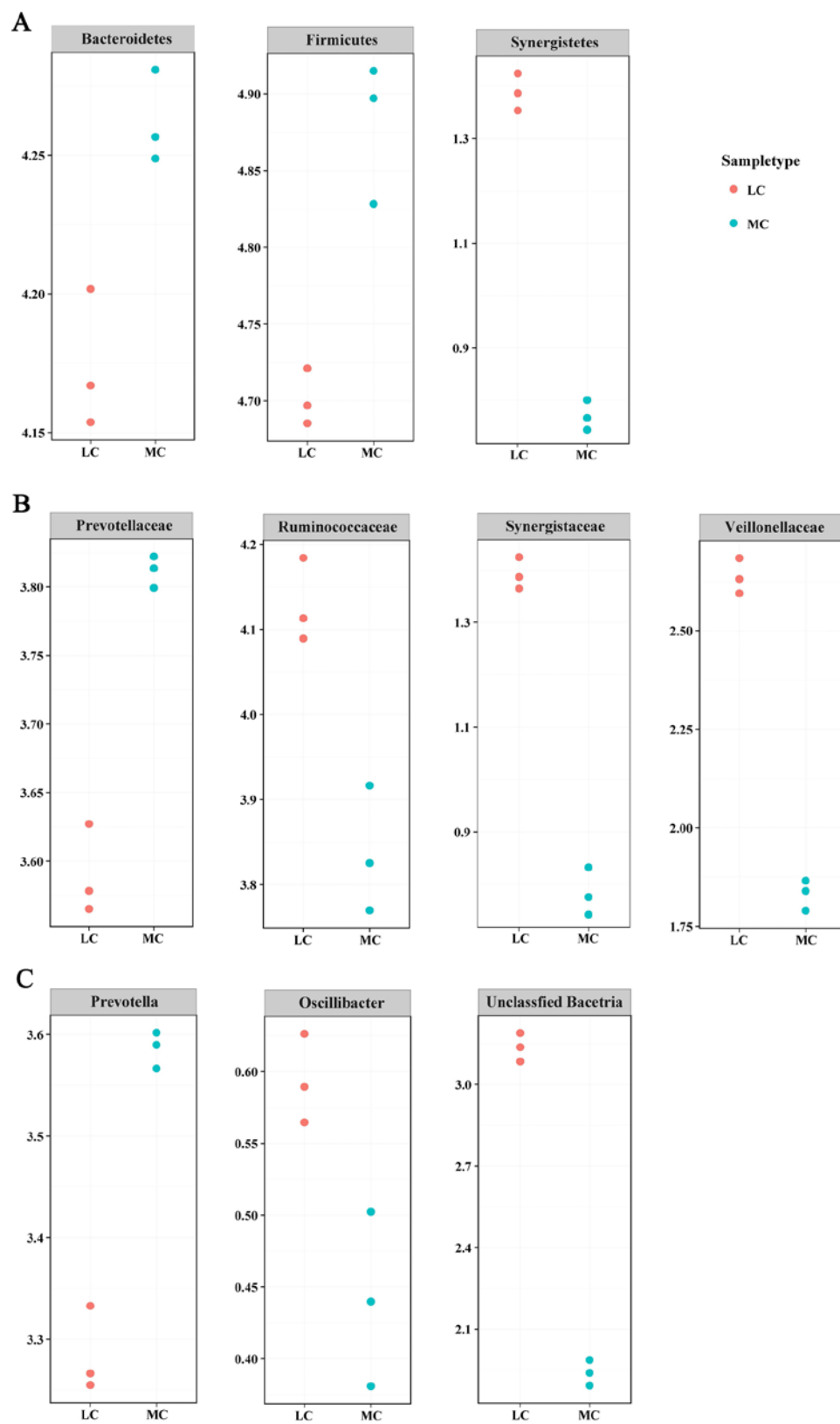

Supplement: Supplementary file 4 — Diversity of the prokaryotic taxa estimated by using the Shannon index. (PDF 128 kb) [file 40168_2017_341_MOESM4_ESM.pdf]
