# Supplementary material for: Associations among dietary non-fiber carbohydrate, ruminal microbiota and epithelium G-protein-coupled receptor, and histone deacetylase regulations in goats
Source: Microbiome. 2017 Sep 19;5:123. doi: 10.1186/s40168-017-0341-z (PMC5606034; doi:10.1186/s40168-017-0341-z)
Supplement: Supplementary file 5 — Expression profile of the members of the GPR family observed in present study. (PDF 14 kb) [file 40168_2017_341_MOESM5_ESM.pdf]

Table S3. Expression profile of the members of the GPR family observed in present study.

Item	Gene	MC (RPKM)	LC (RPKM)	*P*	Chromosome
1	GPR87[1]	111.53±11.92	54.57±4.42	0.008	1
2	GPR1[1]	20.47±2.76	10.94±3.17	0.017	2
3	GPR89A[1]	14.27±1.84	8.42±1.37	0.046	3
4	GPR155[1]	10.28±3.26	4.22±1.36	0.002	2
5	FFAR4[2]	3.80±1.42	10.20±4.44	0.001	26
6	HCAR2[2]	2.32±0.87	8.26±6.86	0.001	17
7	GPR107[2]	1.31±0.38	2.90±0.50	0.008	11
8	GPR160[3]	32.14±4.26	31.78±2.31	0.962	1
9	GPR108[3]	13.33±1.89	19.20±3.11	0.142	7
10	GPR180[3]	8.07±2.11	6.38±1.23	0.442	12
11	GPR137B[3]	5.77±1.79	4.59±0.95	0.372	28
12	GPR171[3]	3.95±0.93	2.11±0.61	0.704	1
13	GPR34[3]	2.95±0.90	0.78±0.28	0.130	X
14	GPR157[3]	2.58±2.58	6.15±1.66	1	16
15	GPR15[3]	2.49±1.07	1.52±0.54	0.255	1
16	GPR19[3]	2.27±0.56	1.84±0.68	0.616	5
17	GPR18[3]	2.05±0.69	1.41±0.28	0.709	12
18	GPR65[3]	1.72±0.48	0.77±0.53	0.083	10
19	GPR82[3]	1.19±0.58	0.41±0.07	0.397	X
20	GPR137[3]	1.12±0.73	1.06±0.21	0.893	29

Values are mean ± standard error of mean (SEM).

P is the *p* value obtained from the two-side t-test.

[1] indicated the genes significantly upregulated in the MC group, compared with the LC group.

[2] indicated the genes significantly downregulated in the MC group, compared with the LC group.

[3] indicated the genes expressed in the rumen epithelium, but no significant difference.
