# Supplementary material for: Associations among dietary non-fiber carbohydrate, ruminal microbiota and epithelium G-protein-coupled receptor, and histone deacetylase regulations in goats
Source: Microbiome. 2017 Sep 19;5:123. doi: 10.1186/s40168-017-0341-z (PMC5606034; doi:10.1186/s40168-017-0341-z)
Supplement: Supplementary file 6 — Expression profile of the members of the HDAC family observed in present study. (PDF 10 kb) [file 40168_2017_341_MOESM6_ESM.pdf]

Table S4. The expression profile of the members of the HDAC family observed in present study.

Item	Gene	MC (RPKM)	LC (RPKM)	*P*	Chromosome
1	HDAC1[1]	215.33±37.70	173.88±5.53	0.033915	2
2	HDAC5[2]	6.01±2.56	12.32±1.47	0.0071	19
3	HDAC6[2]	1.80±0.31	3.52±0.54	0.0298	X
4	HDAC4[2]	2.25±1.14	4.39±2.30	0.0444	3
5	HDAC10[2]	1.38±0.71	3.06±0.22	0.04435	5
6	HDAC3[3]	26.89±5.04	25.67±1.16	0.844	7
7	HDAC8[3]	10.59±2.41	8.35±1.43	0.38155	X
8	HDAC2[3]	37.70±2.44	37.93±4.44	0.9767	9

Values are mean ± standard error of mean (SEM).

P is the *p* value obtained from the two-side t-test.

[1] indicated the genes significantly upregulated in the MC group, compared with the LC group.

[2] indicated the genes significantly downregulated in the MC group, compared with the LC group.

[3] indicated the genes expressed in the rumen epithelium, but no significant difference.
