# Supplementary material for: Associations among dietary non-fiber carbohydrate, ruminal microbiota and epithelium G-protein-coupled receptor, and histone deacetylase regulations in goats
Source: Microbiome. 2017 Sep 19;5:123. doi: 10.1186/s40168-017-0341-z (PMC5606034; doi:10.1186/s40168-017-0341-z)

Fig. S3. Maximum likelihood trees constructed by using the GPR sequences resulting from blast searching in the NCBI nucletide collection with the threshold of the e-value less than 1E-5.

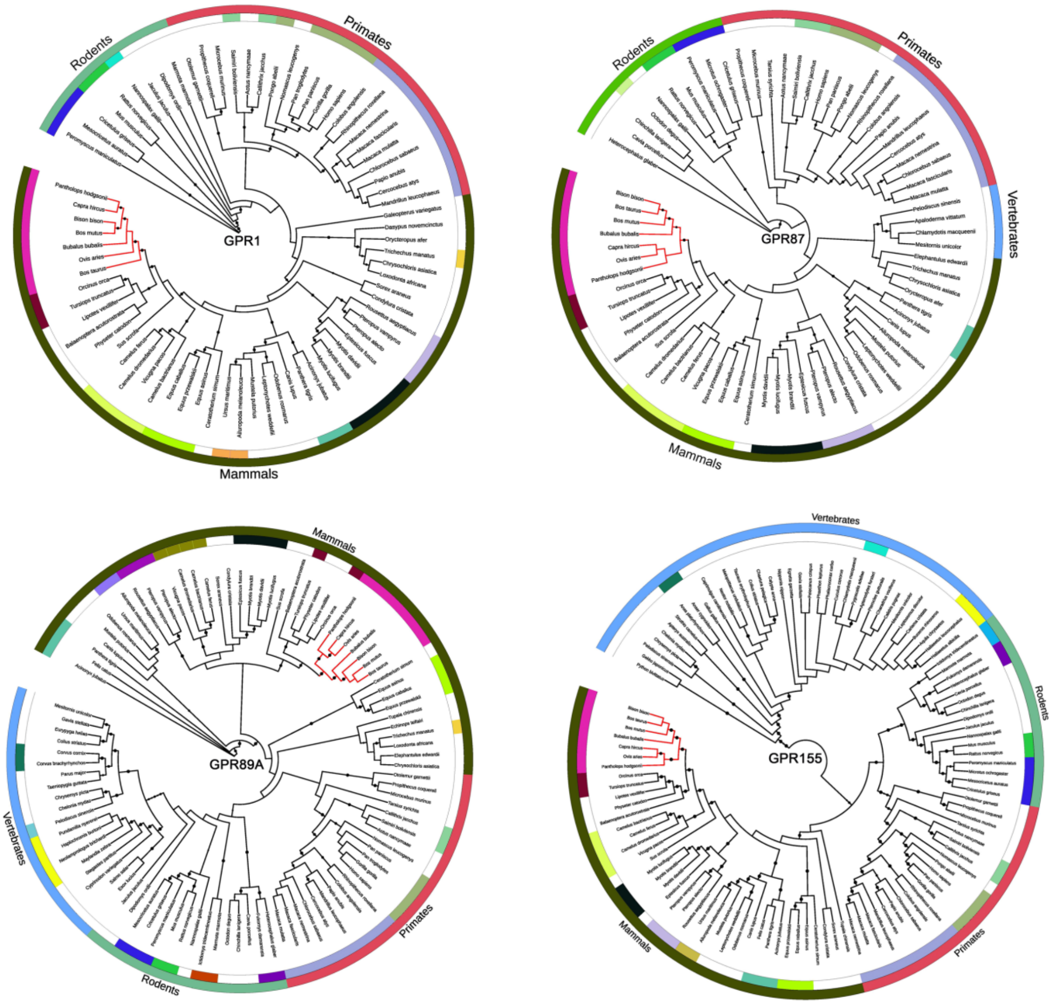

Supplement: Supplementary file 7 — Maximum likelihood trees constructed by using the GPR sequences resulting from blast searching in the NCBI nucleotide collection with the threshold of the e value less than 1E−5. (PDF 770 kb) [file 40168_2017_341_MOESM7_ESM.pdf]
