# Supplementary material for: Associations among dietary non-fiber carbohydrate, ruminal microbiota and epithelium G-protein-coupled receptor, and histone deacetylase regulations in goats
Source: Microbiome. 2017 Sep 19;5:123. doi: 10.1186/s40168-017-0341-z (PMC5606034; doi:10.1186/s40168-017-0341-z)
Supplement: Supplementary file 9 — Other possible functions of the first neighbors connected to the differentially expressed GPRs and HDACs in the network. (PDF 63 kb) [file 40168_2017_341_MOESM9_ESM.pdf]

Table S5. Other possible functions of the first neighbors connected to the differentially expressed GPRs and HDACs in the network.

Gene	Target	KEGG_Level_I	KEGG_Level_II	Function
GPR1[1]	ELK1[1]	Cellular Processes	Focal adhesion	
	ELK1[1]	Environmental Information Processing	ErbB signaling pathway	Angiogenesis, Migration, Invasion
		Endocrine system	GnRH signaling pathway	Gene Expression
		Environmental Information Processing	Ras signaling pathway	Gene Expression
GPR87[1]	SCP2[1]	PPAR signaling pathway	Endocrine system	
GPR89A[1]	PIK3C2G[1]	Environmental Information Processing	Phosphatidylinositol signaling system	Endocytosis
	SLC3A2[2]	Transporters	Heavy subunits of the heteromeric amino acid transporters	
		Digestive system	Protein digestion and absorption	
HDAC1[2]	MAOA[1]	Nervous system	Dopaminergic synapse	Neuro
		Nervous system	Serotonergic synapse	Neuro
	SLC27A3[2]	Transporters	Fatty acid transporter	Solute Carrier Family (SLC)
HDAC4[1]	PLCG1[2]	Environmental Information Processing	HIF-1 signaling pathway	Increase Oxygen Delivery; Reduce Oxygen Consumption
		Immune system	Natural killer cell mediated cytotoxicity	
		Immune system	Fc epsilon RI signaling pathway	
		Immune system	Fc gamma R-mediated phagocytosis	
		Immune system	Leukocyte transendothelial migration	

Gene	Target	KEGG_Level_I	KEGG_Level_II	Function
		Sensory system	Inflammatory mediator regulation of TRP channels	
	SLC9A3[2]	Digestive system	Bile secretion	
		Digestive system	Mineral absorption	
		Digestive system	Protein digestion and absorption	
		Excretory system	Proximal tubule bicarbonate reclamation	
		Transporters	Na+/H+ exchanger	
HDAC5[1]	PVRL1[2]	Environmental Information Processing	Cell adhesion molecules (CAMs)	
		Cellular Processes	Adherens junction	
	VAV2[2]	Cellular Processes	Regulation of actin cytoskeleton	
		Cellular Processes	Focal adhesion	
		Immune system	Natural killer cell mediated cytotoxicity	
		Immune system	Fc epsilon RI signaling pathway	
		Immune system	Fc gamma R-mediated phagocytosis	
	VAV2[2]	Environmental Information Processing	cAMP signaling pathway	Cytoskeletal Remodeling
		Immune system	Leukocyte transendothelial migration	Cell Motility
		Immune system	Chemokine signaling pathway	Regulation of actin cytoskeleton
		Immune system	T cell receptor signaling pathway	Regulation of actin cytoskeleton
		Immune system	B cell receptor signaling pathway	Regulation of actin cytoskeleton
	ARHGAP35[2]	Immune system	Leukocyte transendothelial migration	Regulation of actin cytoskeleton
		Cellular Processes	Regulation of actin cytoskeleton	

Gene	Target	KEGG_Level_I	KEGG_Level_II	Function
		Cellular Processes	Focal adhesion	
		Immune system	Platelet activation	Inhibit the Platelet Spreading
	RAB11FIP1[2]	Cellular Processes	Endocytosis	
	PRKD3[1]	Endocrine system	Aldosterone synthesis and secretion	
HDAC10[1]	SPHK2[2]	Immune system	Fc gamma R-mediated phagocytosis	
	CD81[2]	Immune system	B cell receptor signaling pathway	Ig Production
	MARS2[2]	Genetic Information Processing	Aminoacyl-tRNA biosynthesis	

[1] indicated the expression of the gene is significant upregulation in the MC group, compared with that in the LC group.

[2] indicated the expression of the gene is significant downregulation in the MC group, compared with that in the LC group.
